# Ultralong organic afterglow from small molecular host-guest materials: state of the art

**DOI:** 10.1038/s41377-025-01954-3

**Published:** 2025-08-27

**Authors:** Yuxin Xiao, Mingyao Shen, Chin-Yiu Chan, Tao Yu, Wei Huang

**Affiliations:** 1 Frontiers Science Center for Flexible Electronics, Xi’an Institute of Flexible Electronics & Xi’an Institute of Biomedical Materials and Engineering, Northwestern Polytechnical University, Xi’an, 710072 China; 2 Key Laboratory of Flexible Electronics of Zhejiang Province, Ningbo Institute of Northwesterm Polytechnical University, Ningbo, 315103 China; 3 Department of Materials Science and Engineering, City University of Hong Kong, Kowloon, Hong Kong SAR China; 4 Department of Chemistry, City University of Hong Kong, Kowloon, Hong Kong SAR China; 5 Key Laboratory of Flexible Electronics & Institute of Advanced Materials Nanjing Tech University, Nanjing, 211816 China; 6 State Key Laboratory of Flexible Electronics (LoFE) & Institute of Advanced Materials, Nanjing University of Posts and Telecommunications, Nanjing, 210023 China

**Keywords:** Polymers, Photonic devices

## Abstract

Ultralong organic afterglow materials are being actively explored as attractive candidates for a wide range of applications such as data storage, security inks, emergency lighting, etc., due to their unique long-lived excited state properties and inherent advantages of low cost, appreciable functionality and ease of preparation. In the last three years, much effort has been devoted to achieving efficient ultralong afterglow from organic small molecules, which possess controllable intermolecular interactions and defined energy levels, making them a good platform to suppress the non-radiative decays, hence stabilizing the excitons for efficient afterglow emissions at room temperature. Nevertheless, there has been a lack of reviews on how efficient ultralong organic afterglow can be systematically achieved from small molecular host-guest materials, which is not conducive to the development of the field. In this review, we have outlined and summarized small-molecule ultralong organic afterglow materials based on different emission mechanisms. We have included emission mechanisms involving ultralong room-temperature phosphorescence (URTP), ultralong thermally activated delayed fluorescence (UTADF) and organic long persistent luminescence (OLPL), where the latter two mechanisms have rarely been reported. In addition, challenges and future perspectives are discussed to emphasize the future directions.

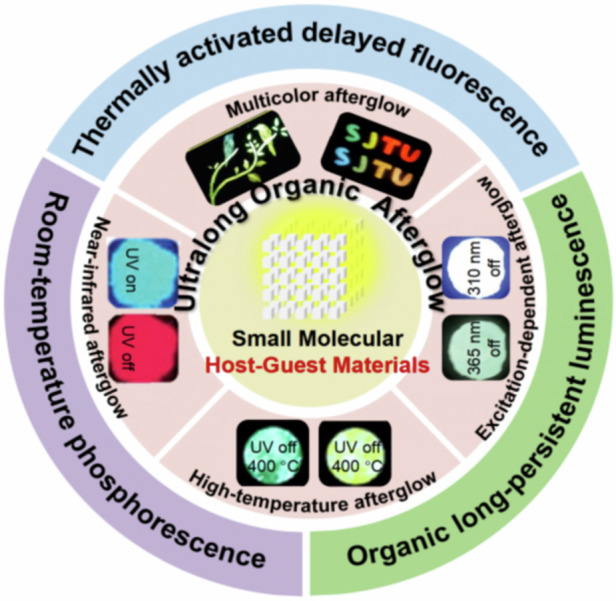

## Abstract (web summary)

In this review, we have summarized ultralong organic host-guest afterglow materials, their emission mechanisms, and discusses future challenges and research directions for these materials.

## Introduction

Ultralong afterglow material is typically used to describe a class of light-emitting materials that can continuously emit light for several seconds to several hours, after the removal of the photo-excitation source^1–5^. Based on its fundamental composition, the ultralong afterglow material can be classified into two categories: organic and inorganic. Inorganic ultralong afterglow materials, leveraging their exceptional persistent luminescence properties, have been extensively implemented in numerous applications ranging from information encryption^6,7^ to display technologies^8,9^, with additional demonstrations in bioimaging and optoelectronic devices^10–13^. However, these inorganic materials have the inherent problems of high production cost, poor processibility and high toxicity. Such characteristics fundamentally restrict their deployment in bio applications. Consequently, the research and development of ultralong organic afterglow materials represent a significant opportunity for advancing societal development and progress.

The field of ultralong organic afterglow materials has recently gained significant attention, deriving from their unique advantages such as tunable emission profiles via large Stokes shifts, exceptional operational lifetimes and rich excited-state dynamics^14–20^. These materials are cost-effective and readily accessible, exhibiting biocompatibility, simple to synthesize, and straightforward to modify. Besides, they possess notable advantages in terms of low toxicity. A number of common design strategies for ultralong organic afterglow materials have been developed nowadays. Among these, crystallization, H-aggregation, and host-guest doping strategy have been identified as promising approaches^21–25^. Notably, the host-guest doping strategy has attracted a significant interest in recent years and has emerged as a prominent and efficient approach for the development of highly efficient ultralong organic afterglow materials.

The host-guest doping strategy is defined as a method which enhances or introduces novel properties to the host system by doping a guest material into it^26–30^. The formation of rigid networks through reversible weak non-covalent interaction forces, such as hydrogen bonding, electrostatic interactions, and Van der Waals forces, between the host material and the guest material results in the restriction of molecular motion and hindrance to non-radiative decay, thus preventing quenching from the surrounding environment and allowing the materials to achieve ultralong organic afterglow emission^31–40^. The host materials can be divided into small molecule-type host and polymer-type host material according to its molecular weight. Small-molecule organic systems exhibit distinct advantages over polymeric materials, including streamlined synthesis pathways, reduced production costs, and precisely tunable excited-state characteristics. These inherent benefits make them ideal model systems for elucidating fundamental photophysical processes with clear energy levels. In general, two principal methods are employed for small molecular host-guest doping strategy, namely solution evaporation method^41–44^ and melt-casting method^45,46^. The solution evaporation approach involves co-dissolving luminescent guest and host materials in a common solvent system under controlled temperature conditions, followed by progressive solvent removal via rotary evaporation. On the other hand, melt-casting method is done by heating the host-guest solid mixture to a homogenous liquid and then cooling it down to form host-guest doped long afterglow material.

In this review, we focus on the recent advances in ultralong organic afterglow materials based on small molecular host-guest doping materials in the last three years and summarize their unique structures and efficient ultralong afterglow properties based on the different emission mechanisms, such as ultralong room-temperature phosphorescence (URTP), ultralong thermally activated delayed fluorescence (UTADF) and organic long persistent luminescence (OLPL). Furthermore, we introduce various ingenious strategies to generate ultralong organic afterglow. Additionally, applications of ultralong organic afterglow materials in various research fields, including advanced information storage, encryption and anticounterfeiting, afterglow display, temperature sensing are also discussed in detail. Finally, some outlooks are given to highlight the opportunities and challenges of ultralong organic afterglow materials to guide the development and design of improved light-emitting materials. This review comprehensively summarized ultralong organic afterglow systems, encompassing not only URTP-based small-molecule materials but also extending to less-reported UTADF- and OLPL-based small-molecule materials. In fact, the latter part represents emerging directions in ultralong organic afterglow materials research^8,11,22,23,29,30,35,45^. We believe this review will serve as a helpful guideline for understanding the luminescent mechanism and design strategies of ultralong organic afterglow materials for achieving high-performance ultralong afterglow systems.

## Luminescence mechanism

**Fig. 1:**
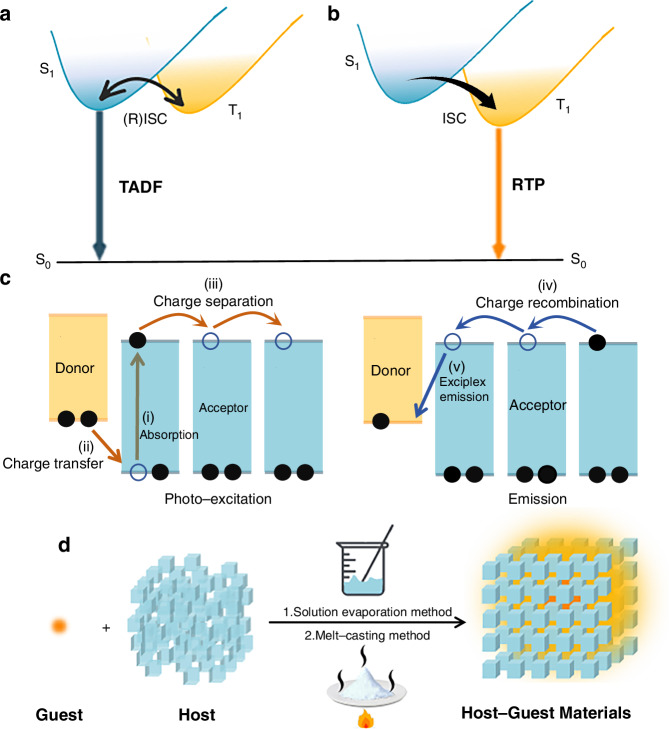
Emission mechanism diagrams of ultralong organic afterglow materials. **a** Schematic diagrams of TADF. **b** Schematic diagrams of RTP. **c** Schematic diagrams of OLPL. **d** Schematic diagrams of principal methods for constructing small molecular host-guest materials

Ultralong organic afterglow materials usually refer to the luminescent materials with emission lifetimes greater than 0.1 s. Such materials based on the host-guest strategy according to the origin of the ultralong afterglow can usually be divided into URTP-based materials, UTADF-based materials and exciplexes based OLPL materials as shown in Fig. 1. In general, an effective energy transfer process, a suppressed non-radiative decay process and a good intersystem crossing (ISC) process from the host to the guest are the keys to build efficient ultralong organic afterglow system from small molecular host-guest materials.

Normally, the luminescent material is excited from the ground state (S_0_) to the singlet excited state (S_n_) under light irradiation, producing singlet excitons in this process. Then, under the effect of large spin-orbit coupling (SOC), part of the singlet excitons are converted into the triplet excited state (T_n_) or the lowest triplet excited state (T_1_) through the ISC process. Occasionally, the triplet excitons can be converted back to S_n_ state through the reverse ISC (RISC) process, if the energy gap between triplet and singlet excited states is small, hence emitting delayed fluorescence from S_1_. Although there is a significant difference in the lifetimes of prompt fluorescence and delayed fluorescence, the prompt and delayed fluorescent spectra largely overlap because their emission origins are from S_1_. Thermally activated delayed fluorescence (TADF) materials generally consist of electron acceptors (A) and donors (D), which result in the separation of highest occupied molecular orbital (HOMO)/lowest unoccupied molecular orbital (LUMO), thereby a small energy gap between S_1_ and T_1_.

On the other hand, there is an alternative radiative pathway that triplet excitons are directly returned to S_0_, hence emitting phosphorescence; however, under ambient conditions, non-radiative decay pathways like molecular rotations, vibrations and intermolecular collisions, such as oxygen molecules, can rapidly quench the triplet excitons. Considering the energy levels, it has been found that a large energy gap between S_1_ and T_1_ states of the most organic molecules is not conducive to the ISC process. Therefore, only a few URTP emissions can be observed in single-component system by the naked eyes. URTP materials with excellent performances generally need the following requirements: (1) an efficient ISC process between S_1_ and T_1_; (2) a high radiative decay rate of the T_n_ excitons; and (3) a suppressed deactivation process (non-radiative decay) of the T_n_ excitons.

In addition, it has been shown that organic D-A system can also emit light with long lifetimes after photoinduced ionization followed by charge separation (CS) process. Such emission mechanism is called exciplexes based OLPL. The photoinduced ionization state is generally associated with two-photon absorption, where the first absorption of photons produces a charge-transfer excited state, and the second absorption of photons, which has an energy in excess of the ionization potential, creates a charge-separated (ionized) state. The two-photon ionization process generally requires a very strong excitation source at a very low temperature; however, recent studies have shown that the charge-separated state that can also be formed in the presence of weak light radiation^47^. Shining light to D-A system, an electron is excited from the HOMO of donor to its LUMO, followed by an electron transfer from the donor’s LUMO to the acceptor’s LUMO, hence forming a charge-transfer state and eventually splits into a charge-separated state as shown in Fig. 1c.

## Research progress

### Room-temperature phosphorescence (RTP) based ultralong organic afterglow materials

Recently, more and more researchers focus on purely organic URTP systems owing to their outstanding solvent compatibility and the precise control of excited-state dynamics. Among them, commercial carbazole (Cz), which has been widely used in building monocomponent organic functional materials, plays a vital molecular building block for RTP, TADF, organic radical luminescence, and organic semiconductor lasers^48–50^. However, in 2020, Liu and co-workers discovered that doping low concentrations (<0.5 mol%) of heterogeneous Cz to Cz resulted in significant afterglows, whereas pure Cz did not exhibit afterglow^2^. Since then, researchers have realized the importance of using trace amounts of guest for the construction of effective ultralong organic afterglow materials. The heterogeneous Cz, that is 1H-benzo[f]indole (Bd), has raised a great concern, since Bd was found to significantly affect RTP in long afterglow luminescence. With reference to this finding, Huang, Chi, Yang and co-workers developed a series of host materials to study the role of Bd on achieving RTP in 2022^51^. In these systems, Bd was introduced as a guest into different organic matrixes including substituted Cz derivatives, e.g., BrPMe. The phosphorescence at 560–620 nm was confirmed to be attributed to Bd itself, which can be detected in regardless of whether Bd is doped in the crystalline or amorphous state of the Cz derivatives. Moreover, the phosphorescent lifetimes of Bd in all these doped systems (even in the amorphous Cz derivatives) were over 0.1 s with yellow afterglow lasting for over 0.5 s, which can be observed by naked eyes. This is the first demonstration of the utilization of Bd as the guest for URTP.

In addition, addressing the scientific debate surrounding boric acid’s RTP capabilities, systematic investigations by Gierschner, Marder and co-workers revealed that purified BA crystals did not exhibit RTP properties under ambient conditions, thereby providing strong evidence that some long-lived emissions are due to impurities^52^. At the same year, Jiang, Lin and co-workers reported a series of color-controllable RTP materials by controlling the pyrolysis temperatures via adapting 1-(2-hydroxyethyl)-urea (H-urea) as guest and BA as host matrix^53^. The host-guest materials were prepared by a direct pyrolysis of a mixture of H-urea and BA at different temperatures of 240, 260 and 300 °C. Interestingly, cyan, green, and yellow afterglows were observed from three materials, after the excitation source was turned off. They proposed that the tunable RTP colors originate from the formation of multiple luminescent centers embedded in situ in the matrix during the pyrolysis process. In addition, the average afterglow lifetimes of three materials were calculated to be 0.95–1.32 s, indicating their long-lived RTP features.

**Fig. 2:**
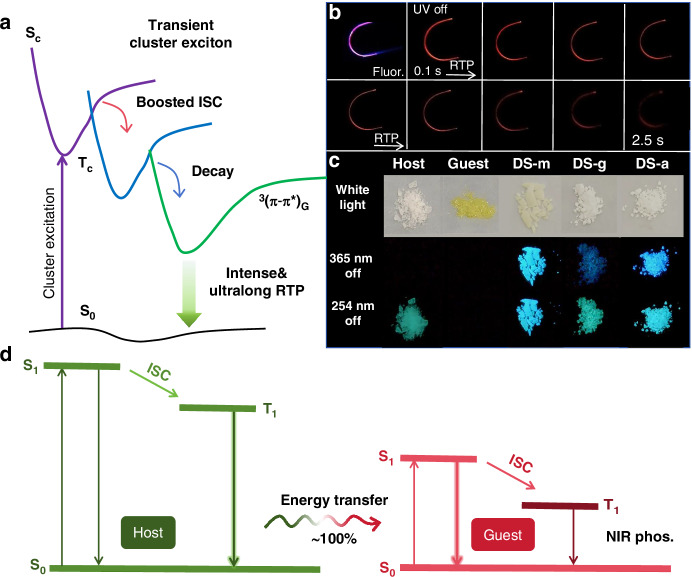
Strategies for achieving URTP in ultralong organic afterglow materials. **a** Schematic illustration of cluster exciton in doping materials for RTP emission^54^. Reproduced with permission^54^. Copyright 2022, Elsevier. **b** Photographs of Py-coated flexible crystals under 365 nm excitation and after 365 nm irradiation^55^. Reproduced with permission^55^. Copyright 2022, John Wiley and Sons. **c** Images of host, guest, DS-a, DS-g and DS-m with doping ratio of 1:1000 before and after 365/254 nm UV irradiation under ambient conditions^65^. Reproduced with permission^65^. Copyright 2023, Springer Nature. **d** Jablonski diagram illustrating RTP mechanisms in organic visible and NIR emitting host-guest materials^78^. Reproduced with permission^78^. Copyright 2024, John Wiley and Sons

Tang and co-workers unveiled host-guest afterglow materials by utilizing long-lived triplet excitons of naphthalene (NL). The triplet excitons of NL were generated from an enhanced ISC process that is caused from different host materials, including 1,4-dichlorobenzene (DCB), 1,2,4,5-tetrachlorobenzene (TriCB), 4-dimethoxybenzene (DMB) and 1,2,3-trichlorobenzene (TriCB)^54^. Doping NL in DCB, i.e., NL@DCB, showed a high RTP quantum yield of >20% and an ultralong lifetime of >0.76 s (duration ~10 s) simultaneously at ambient conditions. The calculations were performed to understand the mechanism, which verified the importance of the matching of the energy levels between the guest and the host. Overall, the cluster excitons lead to large SOC constants and increase the number of effective ISC channels to bridge singlet and triplet excited states and finally result in enhanced RTP as illustrated in Fig. 2a. Consequently, the two-component RTP originates from the impurities as the “triple-state trap.” This proposed mechanism offers an effective approach to harvest triplet states by circumventing ISC.

Various intermolecular interactions exist in the host-guest system, which could be useful for constructing flexible materials. Ouyang. Zhang and co-workers demonstrated flexible organic single crystals with evident RTP via the host-guest strategy^55^. The resulting single-stranded crystal displays stimuli-response properties with a high degree of flexibility. Upon the removal of the stressor, the flexible crystal can rapidly revert to its original straight configuration together with a bright and intense red RTP. The theoretical and experimental results demonstrate that the bright RTP for up to 2.5 s after the removal of 365 nm radiation source arises from the Förster resonance energy transfer (FRET) from the triphenylene (TPhE, host) to the Pyrene (Py, guest) as shown in Fig. 2b. This strategy offered a novel and universal approach for the preparation of flexible organic single crystals with RTP. More recently, Liu and co-workers reported the design of four Cz derivatives, namely PyCz, MPyCz, BPCz and MBPCz, with different extended conjugation lengths that are used as dopants to achieve URTP emissions with lifetimes of over 0.7 s in deep red and near-infrared (NIR) egions. Additionally, these doping systems can be excited by visible light to achieve sustained phosphorescence, providing a promising strategy for the development of visible light-photoexcited ultralong organic afterglow materials^56^.

In addition, host-guest doping strategy could be an effective strategy for constructing smart excitation-dependent (Ex-De) ultralong organic afterglow systems. In 2023, Liu and co-workers prepared RTP materials based on Ex-De strategy by doping 4-carboxyphenylboronic acid (CPBA) acid into the BA^57^, whose RTP can be achieved by exciting the materials with a ultraviolet (UV) light source at 310 and 365 nm for dark blue and green phosphorescence emission, respectively. The doping materials showed a green afterglow visible to naked eyes for ≈10 s after 365 nm UV excitation has stopped, and a dark blue afterglow visible to naked eyes for about 25 s after 310 nm UV excitation has stopped. Furthermore, Xu, Fu and co-workers developed a host surface-induced strategy to achieve Ex-De afterglow emission by doping PCz into dimethyl terephthalate (DTT) materials as PCz@DTT system, which showed a green RTP afterglow more than 6 s with a lifetime of up to 1.0771 ± 0.015 s under ambient conditions^58^. Moreover, the green afterglow can be selectively turned on/off by changing the excitation light from 254 to 365 nm, which could be further applied in advanced information encryption using fluorescence and phosphorescence patterns. The RTP materials based on Ex-De strategy are mainly derived from the guest materials.

Ultralong organic afterglow materials can also be used for developing higher-resolution afterglow imaging. However, increasing the excitation intensity for generating brighter afterglow emission decreases the resolution of afterglow images. Hirata and co-workers reported an amorphous *β*-estradiol film doped with 3 weight percentage (wt%) chromophore 1 (Ch1), which demonstrated an improved resolution of RTP afterglow emission using the triplet depletion^59^. As a result, after the 360 nm light ceased, Ch1 generated a green RTP with an average lifetime of 1.0 s. It has been postulated that the depletion beam-induced ionization from T_1_ results in the formation of a charge-separation state between the guest and host molecules. Subsequently, upconverted delayed fluorescence occurs via the recombination of the charges generated under the depletion beam, which has been observed as a process whereby the long-lived triplet excitons return to S_0_.

Due to the ultrafast non-radiative decay from the liquid medium, there has been limited works on the development of solution-phase ultralong organic afterglow materials. However, the development of ultralong organic afterglow materials in the aqueous phase is of great importance for their potential medical and biological applications^60^. Yang, Tang, Li and co-workers demonstrated a co-assembly of *p*-biphenylboronic acid (*p*-Bph-BOH, guest) with *β*-cyclodextrin (*β*-CD, host) in water to construct ultralong organic afterglow materials^60^. After the sonication for 20 min, the guest molecules are embedded into the host cavity, resulting in RTP with a long lifetime of 1.03 s in water. The intermolecular interactions, including hydrogen bonds, between *β*-CD and *p*-Bph-BOH play an important role to fine-tune the RTP emission. Changing the position of the substituent on boronic acid induced different degree of steric hindrance. It has been found that *o*-Bph-BOH and *m*-Bph-BOH could not be incorporated into the *β*-CD cavity, thus resulting in no RTP emission for *o*-Bph-BOH and *m*-Bph-BOH. In 2023, Hirata and co-workers reported a series of doped crystalline materials to construct high-resolution afterglow patterns^61^. By doping (*S*)-( − )-2,2’-bis(diphenylphosphino)-1,1’-binaphthyl [(*S*)-BINAP] into (*S*)-( − )-2,2’-bis(diphenylphosphino)-5,5’,6,6’,7,7’8,8’-octahydro-1,1’-binaphthyl [(*S*)-H8-BINAP], efficient green afterglow RTP could be observed only in crystalline state. The experimental results showed that RTP of the crystals disappeared under cooperative stimulation with dichloromethane (DCM) vapor and weak photo-irradiation, which was attributed to an induced phase transition from crystalline to amorphous. In addition, the enhanced afterglow intensity enables afterglow detection with individual particles with sizes approaching the diffraction limit in aqueous media. Cucurbit[7] uril (CB7) could also be chosen as a promising host candidate. Biedermann, Brȁse and co-workers utilized the unique host-guest interaction properties of CB7 to achieve selective control over the photolysis reactions of aryl azide in an aqueous medium^62^. In a majority of cases, the photolysis of aryl azides in aqueous solution results in the predominant formation of azepine derivatives through intramolecular rearrangement. However, once this process has happened within the protective confinement of the CB7 cavity, a carboline derivative, namely 4-(2-azidophenyl)-1-methylpyridin-1-ium chloride (1), is formed through C-H amination reaction. As a result, the emitter, carboline 3, is then caged by CB7, which subsequently displays long-lived RTP in the solid state with lifetimes reaching up to 2.1 s.

Researchers developed a series of design strategies to construct host-guest materials, in which Hu and co-workers reported a molecular chaperone strategy to control isolated chromophores to achieve high-performance RTP^63^. The host molecule (DMB) acted as a chaperone molecule, in which different guest molecules are doped to induce its RTP. When guest molecules, such as 4-biphenylamine (BPA), bis(4-biphenyl)amine (BBA), di-2-naphthylamine (DNapA) and N,N′-di-2-naphthyl-1,4-phenylenediamine (DNPD), are non-covalently bound to the DMB, host molecules can prevent the guest molecules from self-aggregation, thereby promoting their crystallinity to suppress non-radiative decay processes. Moreover, the high crystallinity also helps the guest molecules adopt an optimized conformation for the realization of efficient RTP. Eventually, the crystallinity or intermolecular interactions of the chaperone-based host-guest system can be altered by an external force or heat, which, in turn, results in afterglow materials with tunable luminescent properties.

Triphenylamine (TPA) and its derivatives have been extensively employed as host materials for a wide range of host-guest RTP systems due to its great luminescent properties. However, Li et al. first reported that the yellow-green RTP of commercial triphenylamine is induced by the trace amount of naphthyl-substituted TPA impurities^64^. The experimental results also prove that adding trace amount of *N*-diphenylnaphthalen-1-amine (TPA1) or *N, N*-diphenylnaphthalen-2-amine (TPA2) into highly pure lab-synthesized TPA results in RTP, even at an extremely low concentration (1,000,000 : 1, mass ratio). Besides, they also achieved high performance host-guest RTP materials with a long lifetime of RTP of up to 0.290 s (TPA1@TPA) and a high phosphorescence quantum yields of 25.54% (TPP1@triphenylphine(TPP)), respectively, rendering them an attractive option for the encryption of high-security data and the prevention of counterfeiting.

He, Huang, Li, Tang and co-workers provided an efficient strategy to realize Ex-De afterglow in a host-guest system, in which 9(*10H*)-acridone (AD) acted as guest and diphenylsulfone (DS) acted as host as shown in Fig. 2c^65^. The energy transfer process from the higher triplet excited state, i.e., T_2_, of the host to S_1_ of the aggregated/unimolecular guest.

Constructing hydrogen-bonded organic networks has been proved to be a great idea to obtain water-sensitive ultralong organic afterglow materials. Ma and co-workers developed efficient host-guest materials with a water-induced enhancement of URTP, namely CT (a composition of trimesic acid and cyanuric acid) and CTW (incorporating 20 wt.% water into CT)^66^. The incorporation of water in CTW led to an increase of RTP efficiency from 9.3% to 46.1%, as well as an elongated URTP lifetime from 1.13 s to 1.67 s. The results could be attributed to the strengthened intermolecular interactions through the formation of dense and rigid hydrogen-bond networks. This study highlights the mechanism by which water improves the performance of URTP materials, providing a new route for the exploitation of moisture-mediated URTP materials.

Ultralong organic afterglow materials also provide a practical solution to prepare long-wavelength and long-lifetime organic URTP materials for the applications in bioimaging. Cai and co-workers developed a series of pyrrole-based molecules with enhanced molecular conjugation and low triplet energy levels^67^. MAP1@benzophenone (BP) exhibited a visible yellow afterglow, lasting for over 2 s. Yang, Chi and co-workers developed a series of amorphous doping materials by simply thermal annealing using the host (DPOBP-Br) that is integrated of diphenylphosphine oxide (DPO) and 4-bromo-benzophenone. Taking advantages of heavy atom effects and efficient energy transfer processes, it has been found that DPOBP-Br acts as a “triple exciton pump” and facilitates the generation of triple excitons in the guest. Eight commercial dyes with emission color ranging from green to red were doped into DPOBP−Br, including 3,6-diaminoacridine hydrochloride (PF), dibenzofuran (DBF), 1,8-naphthalic anhydride (NAPP), 1,8-naphthalimide (NAPM), 4,4′,4′′,4′′′-(ethene-1,1,2,2-tetrayl)tetrabenzaldehyde (4CHOTPE), coumarin (CM), pyrene (PY), and rhodamine B (RhB). All eight host-guest materials formed transparent films after thermal annealing. After UV irradiation, these materials showed ultralong organic afterglow with green to red colors and a bright afterglow can still be clearly observed with lifetimes ranging from 0.021 s to 0.166 s^68^. Furthermore, controlling the residual oxygen concentration in the amorphous matrix by UV irradiation achieves photo-activated RTP phenomenon, i.e., dynamic RTP. Upon a prolong UV irradiation for 7 s, triplet oxygen has been gradually transformed into singlet oxygen, which reduces the concentration of triplet oxygen, hence activating RTP and causing luminescent changes.

In 2024, Hudson and co-workers developed host-guest afterglow materials by doping *N,N,N,N*-tetrakis(tertbutylphenyl) benzidine (DTBU) into TBBU as DTBU@TBBU^69^. A bright greenish yellow RTP could be observed with a lifetime of 0.340 s under N_2_ atmosphere. Besides, when the doping concentration of DTBU is increased to 10 mol%, the afterglow cannot be quenched by air completely and maintains a long lifetime of 0.179 s under ambient conditions.

DTT has been developed as host materials due to its strong proton acceptor, which could facilitate to modulate the interactions between the host and guest materials. In 2024, Man, Tang, Fu and co-workers have found that it is possible to facilitate the proton migration of the N─H bond of Cz-derived guest materials, where ultralong organic afterglow emissions can be tuned by excitation wavelength or amine stimulation^70^. The host-guest materials exhibit a clear Ex-De emission. When the excitation wavelength is below 300 nm, strong proton interactions with photo-excited DTT result in afterglow emission. On the contrary, when the excitation wavelength is over 300 nm, weak proton interactions with ground-state DTT suppress afterglow emission. Recently, Man, Liao, Tang and co-workers reported a series of guests with atoms having different atomic sizes such as named C, S and O and used them as guest materials doping into DTT as C@DTT, S@DTT and O@DTT. The doping materials exhibited great URTP properties. C@DTT exhibited Ex-De green afterglow emission lasting for 1.5 s under 254 nm light excitation and 0.5 s under 365 nm light excitation; S@DDT showed afterglow emission lasting for 2 s (λ_ex_:254 nm) or 1 s (λ_ex_:365 nm); O@DDT displayed afterglow emission of up to 4.5 s (λ_ex_:254 nm) or 2 s (λ_ex_:365 nm). The experimental results and density functional theory (DFT) results indicate that the afterglow originates from the phosphorescence of guest molecules, and O having a higher SOC value showed a longer lifetime^71^.

In addition to the physical doping strategy, researchers also proposed covalent bond-linked host-guest doping strategy, in which three different aminobenzoic acids are covalently bonded to a cyanuric acid (CA) matrix. Chen, Wang and co-workers developed a binary matrix based on the cyanuric acid (CA) and different amines like melamine (MA), biuret (BU) and urea (UA) as host materials^72^. It is shown that strong interactions between two components of the binary matrix lead to changes in molecular configuration of guest and their excited state distributions, hence promoting more triple excitons. Moreover, the binary matrix also exhibits a stronger confinement effect when compared to the single CA matrix. The stronger confinement effectively suppresses non-radiative decay of triple excitons, in which the emission intensity of the ultralong organic afterglow of the binary matrix could reach 28-fold higher than that of the CA single matrix, together with a lengthened lifetime of 1.51 s. Liu and co-workers proposed a microwave heating strategy to form the covalent bonds between the guest molecule and CA, which provides an additional suppression of molecular vibrations, thereby leading to high-performance ultralong organic afterglow materials with long afterglow lifetimes of up to 0.800 s, high photoluminescence quantum yields (PLQY) of 98.95% and phosphorescence quantum yields of 76.42%^73^. With reference to the findings in CA2-based afterglow materials, Yin, Chen, Cai and co-workers has identify the construction of HOFs by co-assembling melamine (MA) and CA^74^. Taking advantage of rigid microenvironments in HOFs, non-radiative decay from the guest, i.e., 2-naphthoic acid (2-NA), is strongly suppressed, where populated triplet excitons are significantly stabilized by geometrical confinement. The doping materials in aqueous medium displayed URTP emission with an ultralong phosphorescent lifetime of up to 0.4931 s. Additionally, such doping materials were stable in water or even in acidic/basic condition for more than 10 days.

Natural products can also be used for host-guest doping to develop novel ultralong organic afterglow materials. For example, Chen, Zhang and co-workers developed the host-guest doping strategy for the chiral recognition of natural amino acids^75^. The use of long-lived RTP emission for chiral recognition was done by combining benzamide host (F-Ph) with the naphthamide guest (F-Na) at a doping concentration as low as 10 ppm. It has been found that the L-forms of amino acid induce strong RTP, whereas the unnatural D-forms of amino acid produce insignificant RTP. The results broaden the scope of chiral sensing with luminescence by reducing the requirements of specific molecular structures.

Researchers have proved that crystallinity could also play a vital role in constructing ultralong organic afterglow materials. Mei and co-workers achieved ultralong organic afterglow systems featuring with vibration-induced emissions by simply doping butterfly-like guests, namely *N,N*′-diphenyl-dihydrodibenzo[a,c]phenazines (DPACs), into small-molecule hosts like BP, TPP, TPA and triphenylarsine (TPAs)^76^. The critical role of host crystallinity on URTP behavior of host-guest doping systems was first proposed. Dai, Wang, Huang and co-workers firstly introduced 2,3,5-triarylfuro[3,2-b]pyridines with twisted molecular conformations as guest materials and phenyl(pyridin-2-yl)methanone (PPy), which can provide rigid microenvironment, as the host material. After that, doping commercial dyes, such as erythrosin B sodium salt (EB) or eosin Y sodium salt (EY) as energy acceptors into the host-guest systems, the resulting systems exhibited significant red RTP, which could be attributed to efficient FRET processes from T_1H_ of 2,3,5-triarylfuro[3,2-b]pyridines to S_1G_ of commercial dyes^77^.

In 2024, Li, Li and co-workers first developed persistent NIR RTP materials by doping NIR guest materials (G-CS) into a host matrix (Host-CS) to construct G-CS@Host-CS. It has been found that the persistent NIR phosphorescence in host-guest doping system showed a ten-fold enhancement of URTP lifetimes to 492 ms, when compared to that of guest material (G-CS). The enhancement can be explained by the highly efficient energy transfer via reasonable modulation of aggregated structures of host-guest doping systems as shown in Fig. 2d^78^.

### TADF and mixed TADF/RTP long afterglow materials

Emerging as a promising strategy beyond conventional phosphorescent systems, TADF enables triplet-to-singlet exciton conversion via RISC, thereby offering an alternative pathway for achieving ultralong organic afterglow materials. The afterglow from both TADF and RTP emissions of organic materials could be realized by the host-guest doping strategy. In general, mixed TADF- and RTP-afterglow emissions are derived from the intrinsic luminescent properties of guest materials or the intermolecular charge transfer between host and guest. In 2023, Shao, Cai and co-workers innovatively constructed a dynamic and controllable afterglow emission system based an interesting buckybowl molecule, i.e., oxazole-fused (*S, S*-dioxide) trithiasumanenes-chlorobenzene compound (OTC). It has been known that the non-uniform electrostatic potential distributions of polycyclic aromatics can be used for dynamic modulation of transition processes of triplet excitons^79^. Several host materials were selected to provide different intermolecular interactions, which, in turn, achieved a variety of afterglow emissions in the host-guest materials. As a result, the aggregates of OTC were dominant in CPAN and DPAN, and the modulation of RTP and TADF afterglow was achieved by the intrinsic competition between ISC process of OTC-monomer and RISC process of OTC-aggregates processes.

**Fig. 3:**
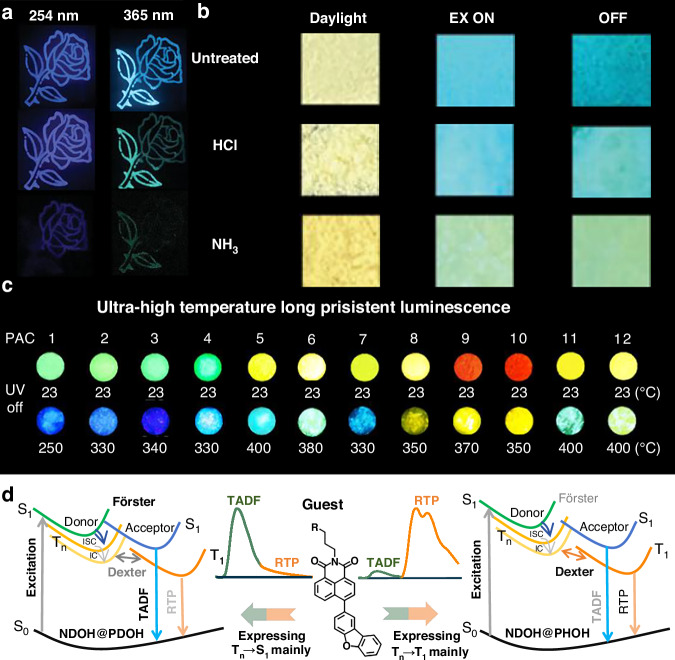
Strategies for achieving UTADF in ultralong organic afterglow materials and representative afterglow photographs. **a** Photos of a flower pattern under the irradiation of 254 and 365 nm UV light, and after switching off the UV light for 2 s^80^. Reproduced with permission^80^. Copyright 2023, John Wiley and Sons. **b** Time-resolved images before and after turning off the 365 nm excitation of untreated, HCl-fumed and NH_3_-fumed FL@BA. Reproduced with permission [ref. ^81^]. Copyright 2024, John Wiley and Sons. **c** Optical images of the resulting B_2_O_3_ crystalloids embedded with 12 types of PACs at high temperatures in air after switching off the 365 nm excitation light^82^. Reproduced with permission^82^. Copyright 2023, American Chemical Society Publications. **d** Schematic diagram of management of triplet exciton transitions to express T_n_ → S_1_ and T_n_ → T_1_^87^ Reproduced with permission^87^. Copyright 2024, Springer Nature

Wang, Li, Zhao and co-workers reported a series of host-guest materials based on BA matrix via dehydration-induced through-space conjugation (TSC)^80^. Heating BA matrixes that were doped with different guests like o-hydroxybenzoic acid (OHA), m-hydroxybenzoic acid (MHA) and p-hydroxybenzoic acid (PHA) exhibited significant blue TADF and RTP afterglow emissions with lifetimes from 0.83 s to 1.59 s. The results showed that the enhanced degree of TSC between B and O atoms contributes to improved photophysical properties, which resulted in tunable multicolor afterglows in the blue-green region as shown in Fig. 3a. Recently, Wu and co-workers proposed a new strategy to construct TADF-based afterglow materials by activating the n-π* transitions in the host-guest system for efficient afterglow luminescence via matrix triggering^81^. Fluorescein (FL) with a twisted molecular structure was used as guest and BA was chosen as matrix to develop the host-guest material, FL@BA. The radiative pathway of FL was clearly changed after doping in BA matrix, in which FL@BA yielded a high afterglow quantum yield of 24% and a long-lasting cyan afterglow duration of >10 s. Such phenomenon can be attributed to the enhanced SOC by the intermolecular and intramolecular interactions in host-guest system. Benefiting from the visible-light absorption properties of guest, a series of multi-color afterglow phosphors, which can be excited by visible or white light, were further successfully constructed. In addition, FL@BA exhibited reversible acid/base-response properties as shown in Fig. 3b. After fumigation with HCl vapor, the luminescence of FL@BA showed negligible change, indicating that the carboxyl group of FL was inert to acid attack. Contrary, when FL@BA was fumed by NH_3_ vapor, a significant color change was found. Shen, Qiu and co-workers developed a series of B_2_O_3_-based materials with multicolor ultralong organic afterglow properties even under a ultrahigh temperature. The B_2_O_3_-based afterglow materials was made by dispersing aromatic derivatives in an aqueous solution of BA, followed by the processes of drying, melting, and dehydrating as shown in Fig. 3c^82^. The crystalline B_2_O_3_ host exhibited a high rigidity and a thermal stability, which provides a strong confinement effect, thus restricting the vibration and rotation of guest molecules even at extremely high temperatures of up to 400 °C. These results also showed that the resulting ultralong organic afterglows of these host-guesting doping materials were originated from a mixture of UTADF and URTP.

Several research have proved that the energy gap between the singlet and triplet states (ΔE_ST_) value of the new charge-transfer state from host-guest interactions not only facilitates efficient RTP, but also enables the conversion of triplet excitons to S_1_ state via RISC, hence resulting in TADF emission. In 2023, Liu and co-workers found that host-guest doping strategy could balance the efficiency of UTADF and URTP^83^. They reported Cz-based host-guest systems that can achieve a long-lived white-light afterglow, either by the excitation of ultraviolet (UV) or visible light. Further investigation showed that the CS state and the exciton recombination of the charge transfer (CT) state directly determine the lifetime of the afterglow. Meanwhile, an efficient RISC process between ^1^CT and ^3^CT was found to be responsible for the TADF properties of the host-guest system. As a result, the lifetime of the TADF was successfully extended, which was comparable to that of RTP. Additionally, Liu and co-workers reported UTADF materials with Cz derivatives by doping the guest (CBP-Bd_2_CN) into the host materials (CBP-2CN, CBP-CN and CBP). It has been found that the UTADF originated from the intermolecular CT that is generated by the interaction between host and guest, while the URTP was from the guest^84^. Besides, the doping materials exhibited abundant ultralong luminescent colors at different delay times and also response to temperature changes thanks to the various decay rates of UTADF and URTP.

Dong, Li and co-workers achieved UTADF materials by doping D-A type fluorophore, i.e., triphenylaminedibenzo[a,c]phenazine (TPA-PTPQ), into TPA, TPA-PTPQ@TPA exhibits dual delayed emissions at 516 and 605 nm with long lifetimes of up to 0.108 and 0.145 s, respectively, which correspond to TADF and RTP afterglow emission. Both TADF and RTP emissions were found to be temperature sensitive, in which a reversible temperature-dependent afterglow characteristic resulted in a substantial spectrum shift of 90 nm, from green, orange and red colors, at different temperatures^85^.

Ma, Wang and co-workers proposed a series of 2-benzylmalononitrile derivatives (TCN-R, R = H, F, Cl) with trace amounts of impurities (BTDA-R, R = H, F, Cl) to construct efficient host-guest materials with long green afterglows of 1.5–3.5 s. After melting process, 1 wt% TCN-H@BTDA-H material presents persistent dual-band ultralong afterglows at 410 nm and 490 nm with lifetimes of 1.056 s and 1.027 s, respectively, which correspond to TADF and RTP emissions, respectively. Experimental results indicated that the TADF and RTP emission origins are from BTDA-R instead of TCN-R^86^.

Zhang, Li and co-workers realized triplet excitons management in a naphthalimide derivative, namely NDOH, by host engineering, where seven phthalimide derivatives were used as host with different energy levels^87^. The intensity ratio (I_TADF_/I_RTP_) between TADF and RTP emissions of different host-guest systems was further monitored to confirm energy transfer mechanisms, i.e., Dexter exchange energy transfer and FRET as shown in Fig. 3d. The experimental results proved that a larger ΔE_ST_ would result in a more efficient transition from T_1_ of host to that of guest. When ΔE_ST_ was >0.07 eV, I_TADF_/I_RTP_ would be <1 and transitions to T_n_ would dominate rather than to S_n_.

### Exciplexes based OLPL materials

**Fig. 4:**
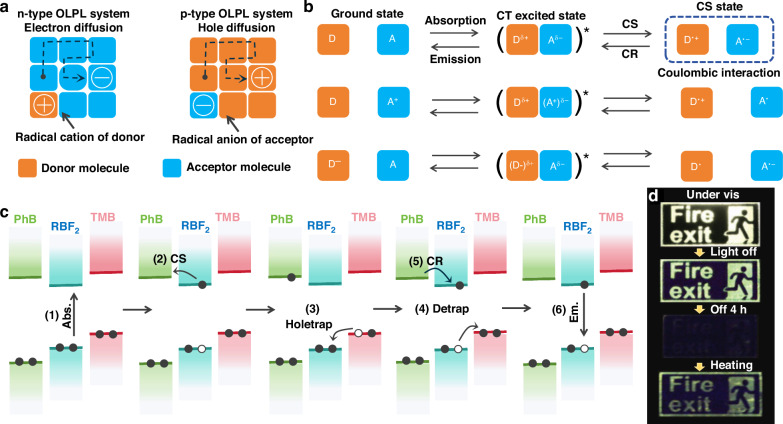
Emission mechanism of OLPL systems. **a** Schematic diagrams of charge-separated states in n-type (left) and p-type (right) OLPL systems^89^. Reproduced with permission^89^. Copyright 2024, Springer Nature. **b** A mixture of neutral donors and acceptors forms radical ion pairs with Coulombic interaction as the charge-separated (CS) state by photoinduced charge transfer (CT) from the ground state, whereas cationic acceptors or anionic donors form neutral radicals. The weaker Coulombic interaction is expected to reduce the probability of charge recombination (CR)^89^. Reproduced with permission^89^. Copyright 2024, Springer Nature. **c** Dual-mechanism design strategy for efficient, long-lived organic afterglow materials^93^. Reproduced with permission^93^. Copyright 2024, American Chemical Society Publications. **d** OLPL materials used for emergency exit signs, which could produce light emission triggered by thermal activation, even after the luminescent intensity becomes nearly invisible^17^. Reproduced with permission^17^. Copyright 2024, John Wiley and Sons

In 2017, Kabe and Adachi developed a class of D-A doping systems for efficient ultralong afterglow emission, which increased the afterglow duration to one hour at one fell swoop (usually ranged from seconds and tens of seconds)^47^. The system consists of an organic electron donor (*N,N,N*′*,N*′-tetramethylbenzidine, TMB) and an organic acceptor (2,8-bis(diphenylphosphoryl)dibenzo[b,d]thiophene, PPT) molecule. The OLPL properties under weak excitation last for more than 1 h, which originates from a photoinduced slow charge recombination from a long-lived charge-separated state. The slow diffusion of free electron carriers generated by the charge separation of charge-transfer exciton is useful for energy storage, which is also responsible for its ultralong afterglow emission. PPT host provides a rigid amorphous environment for the diffusion of free electron carriers, which achieves a long-lived charge-separated state of the D-A doping material to store the absorbed photon energy and leads to a long duration of the OLPL. In addition, the material can be excited by visible light sources and exhibits significant afterglow even at a temperature above 100 °C. By doping various emitters like 2,5,8,11-tetra-tert-butylperylene (TBPe), 9,10-bis[N,N-di-(p-tolyl)-amino]anthracene (TTPA), 2,8-di-tert-butyl-5,11-bis(4-tert-butylphenyl)-6,12-diphenyltetracene (TBRb), tetraphenyldibenzoperiflanthene (DBP), and 4-(dicyanomethylene)-2-methyl-6-julolidyl-9-enyl-4H-pyran (DCM2) to the OLPL system consisting of TMB and PPT, greenish-blue to red and even warm white emissions are achieved^88^. In 2022, they further enhanced the performance of the OLPL material in this system by adding cationic photoredox catalysts as electron-accepting dopants to the neutral electron-donating host, so that the hole diffusion in the doped material would produce a stable CS state as shown in Fig. 4a, b^89^. Moreover, by adding an additional hole-trapping agent, the separation of electrons and holes is significantly stabilized, allowing more holes to be generated, thus the afterglow duration of the film is largely extended by seven times under N_2_ condition.

Liu and co-workers developed a novel D-A doping systems with an electron donor (4,4,4-tris[3-methylphenyl(phenyl)amino]triphenylamine, m-MTDATA) and an electron acceptor (tris-[3-(3-pyridyl)mesityl]borane, 3TPYMB) for excellent OLPL performance. After 1 min of UV irradiation at room temperature, the system exhibits effective OLPL emission that lasted for more than 100 s. Moreover, TADF emission of this system was found to exist prior to OLPL emission. Since the occurrence of RISC and the formation of a CS state, both TADF and OLPL emissions are temperature sensitive^90^.

Jiang, Lin and co-workers developed the first carbon dot (CD)-based OLPL system be facilely and effectively fabricated using a household microwave oven, which exhibited more than 1 h of duration in 2022^91^. Recently, Liu, Liu and co-workers developed OLPL systems by utilizing pure organic CDs, which are formed with isophthalic acid (IPA) and urea, as donors and CA as acceptors. A color-tunable (from blue to green emission) CDs-based OLPL material (CDs@CA) was obtained by a one-step synthesis strategy. This is the first time that color tuning has been achieved in OLPL materials based on CDs. Meanwhile, this work is highly beneficial for developing CDs-based OLPL systems^92^.

In 2024, Zhang and co-workers proposed a dual-mechanism design strategy to achieve an OLPL material along with high afterglow efficiencies by using either RTP or TADF mechanism^93^. They introduced an electron donor as a third component to act as traps and promote CS, and doped intramolecular charge transfer-type difluoroboron *β*-diketone molecules with large S_1_ dipole moments as the luminescent component into organic matrices, which can also achieve a range of TADF-type long afterglows. The obtained materials exhibit a dual afterglow mechanism as shown in Fig. 4c, exhibiting TADF/RTP afterglow with an afterglow efficiency of up to 50.9%, followed by hours-long OLPL afterglow emission with an afterglow efficiency of up to 13.1%.

Guo, Zhang and co-workers developed an efficient OLPL material which could last for several hours under visible light excitation by incorporated a multi-resonance (MR)-TADF molecule into an exciplex system^17^. Aligning the energy levels creates two charge transfer pathways: (1) from the MR-TADF molecule to the acceptor and (2) from the donor to the acceptor, thereby facilitating energy transfer within the composite. The inherent CS characteristics of the opposing resonance effect benefits the requirement of small difference between the local excited and CT states, resulting in the enhanced absorption of the composite material by ten folds. As shown in Fig. 4d, the strategy helped to harvest and store excitation energy more efficiently, resulting in a remarkable OLPL with a duration of over 4 h and the emission could be triggered by thermal activation even after the luminescence intensity has become almost invisible.

Yuan, Huang, Chen and co-workers realized ultralong organic afterglow materials with narrow-band, long-lived, and full-color properties by constructing host materials via isolating MR-TADF emitters in a glassy steroid-type host through melt-cooling method^94^. Exciton dissociation and recombination (EDR) for both long-lived fluorescence and phosphorescence is achieved in the prepared host upon photoirradiation. Efficient FRET from host to various MR-TADF emitters leads to high-performance ultralong organic afterglow, exhibiting a small full width at half maximum (FWHM) of 33 nm, a long persistent time over 10 s, and a facile color-tuning from deep-blue to orange colors (414–600 nm).

### Applications

Given the ultralong afterglow characteristic, host-guest afterglow materials can be used for multi-dimensional information encryption, anti-counterfeiting, information storage, bioimaging and so on. We took some typical examples to illustrate these applications. Ultralong organic afterglow materials continue to emit light even after the light has ceased, and this fundamental property can be exploited for anti-counterfeiting and encryption, among other applications. OLPL materials can be used to construct afterglow displays for emergency lighting applications, as shown in Fig. 4d. Besides, for example as anti-counterfeiting and encryption, the stamp on the paint is invisible under day light^64^. After UV light irradiation, the content of stamps can be seen after light illumination. By thermal mixing of host and guest, the luminescent properties of host-guest afterglow materials can be modified. For example, a thermal printing paper could be designed as following^65^. First, the paper was soaked in the guest solution. Once the solvent had evaporated, fine host powder was smeared on one side of the guest-loaded paper. When the thermal paper passed through the print head of thermal printing machine, guest and host would be partially mixed together to form the doping materials by heat, due to the melting point of host. At 365 or 254 nm excitation, two blue emitting Chinese characters “Shenzhen” were observed with naked eyes after thermal printing^65^.

**Fig. 5:**
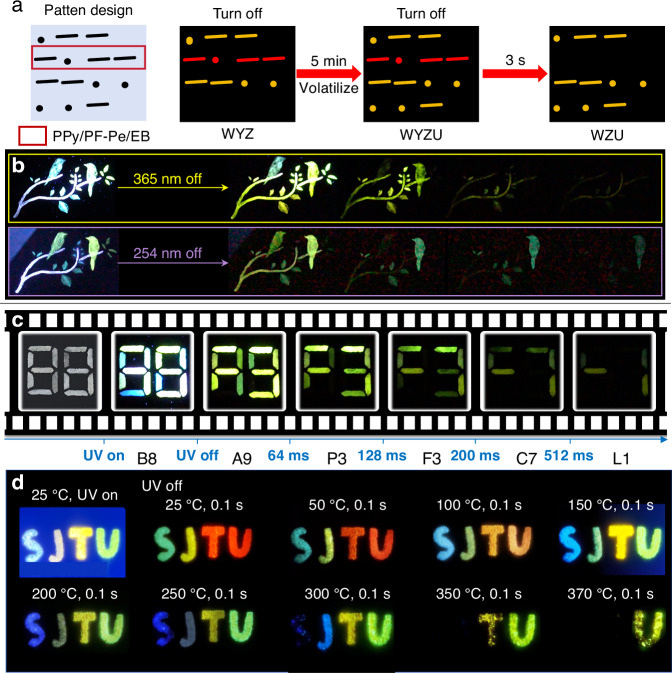
Applications for ultralong organic afterglow materials. **a** Morse code pattern containing different doped materials^77^. Reproduced with permission^77^. Copyright 2024, Elsevier. **b** The photos along with duration time after the stoppage of UV light irradiation with different wavelengths. Reproduced with permission^41^. Copyright 2021, Springer Nature. **c** Photos along with duration time after the stoppage of UV light irradiation with different wavelengths. Reproduced with permission^41^. Copyright 2021, Springer Nature. **d** Temperature-sensitive alphabetic security code derived from PAC1(S)-, PAC7(J)-, PAC10(T)-, and PAC9(U)-embedded B_2_O_3_ crystalloids^82^. Reproduced with permission^82^. Copyright 2024, American Chemical Society Publications

Benefiting from the ultralong lifetime of host-guest afterglow materials, light control can be utilized to develop a range of dynamic anti-counterfeiting methods like Morse codes. As shown in Fig. 5a, the Morse code message in the red dashed box consists of PPy/PF-Pe/EB, while the other Morse code messages consist of PPy/PF-Pe^77^. The Morse code information of all four lines presented after light illumination can be translated as “WYZU”. After the light is switched off for 3 s, the code information of the second line disappears, and the Morse code information of the pattern can be translated as “WZU” because the afterglow time of PPy/PF-Pe/EB is shorter than that of PPy/PF-Pe. By making use of the different afterglow times of different hodt-guest afterglow materials, a specific information can be displayed with respect to time, which demonstrates its progress in the preparation of advanced encryption materials or anti-counterfeiting materials.

Using the colorful emission properties of host-guest afterglow materials, as shown in Fig. 5b, the patterns show different dynamic information after the UV irradiation is stopped at different wavelengths^41^. These ultralong colorful afterglow dynamic response properties, which vary with environmental conditions, can be used for dynamic multi-dimensional anti-counterfeiting. Moreover, with the help of the time-resolved triple exciton decay property, it is also possible to store a considerable amount of optical information in a time range, for example, as shown in Fig. 5c, the information of “B8”, “A9”, “P3”, “P3”, “F3”, “C7” and “L1” could be read at different times over time.

In addition, as the UTADF and URTP show completely opposite trends in response to temperature changes, the doping materials can provide sensing capabilities over a wide temperature range and be utilized as intelligent temperature-sensing or information storage. As shown in Fig. 5d, a temperature sensitive alphabetic security code “SJTU” was first designed^82^. Once stopping the 365 nm light irradiation at an ambient temperature, a colorful afterglow pattern of “SJTU” appeared. With the process of increasing the heating temperature to 370 °C, the character “S”, “J” and “T” disappeared gradually and finally leaving “U” along.

In addition to the applications mentioned above, based on the fact that some of the host-guest doped materials have better NIR phosphorescence properties, organic afterglow materials can be further processed into nanoparticles and applied to bioimaging with deeper penetration and higher signal-to-background ratio^78^. Furthermore, while rigid matrices in crystalline and amorphous state dominate current host-guest systems due to their superior confinement effects and stabilized triplet states, recent advances in aqueous systems and flexible materials show unique potential in applications. Aqueous ultralong afterglow materials, owing to their unique luminescent properties, such as long-lived emission, high stability, and aqueous compatibility, exhibit promising potential for applications in fields like smart displays and information storage, bioimaging and diagnostics, etc. The future applications of flexible ultralong afterglow materials are poised to break through the limitations of traditional rigid materials, driving transformative changes in fields such as flexible electronics, biomedical technologies, and intelligent sensing. With ongoing optimizations in material performance and cross-disciplinary technological integration, ultralong afterglow materials are expected to emerge as a critical branch of next-generation smart materials.

## Summary and outlook

**Fig. 6:**
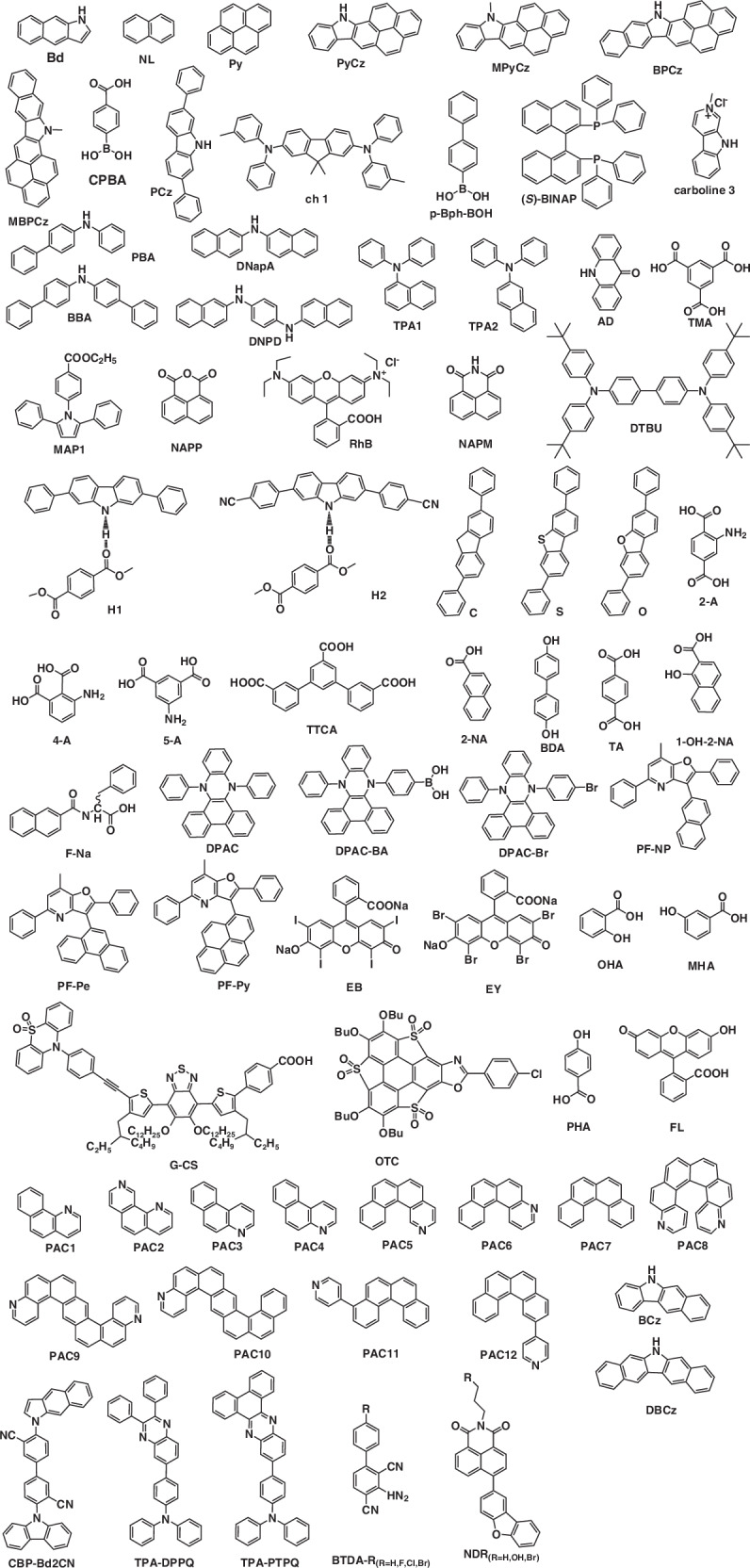
Chemical structures of guest materials

**Fig. 7:**
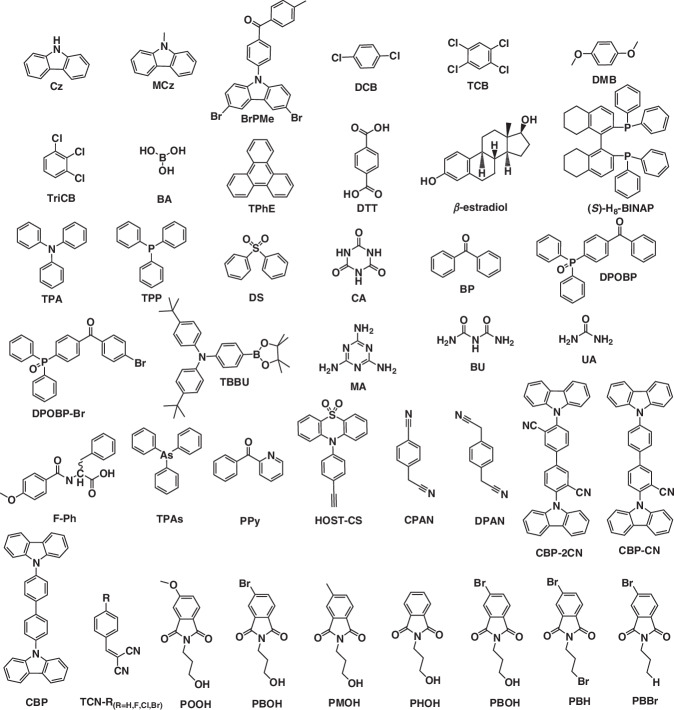
Chemical structures of host materials

The growing interest in purely organic afterglow materials has been driven by the pursuit of high luminescent efficiencies and ultralong emission lifetimes. Researchers have turned to the design of host-guest strategy, where they can take advantage of the rigidity of the host to achieve a confinement effect on the guest to improve afterglow quantum yields and emission lifetimes. Polymer-based ultralong organic afterglow materials have garnered significant attention due to their strong environmental stability, excellent processability, and flexibility. In contrast, small-molecule systems enable precise chemical modifications to tailor the electronic structure of luminescent moieties and optimize intermolecular interactions, thereby achieving fine-tuning of phosphorescence color, lifetime, and stimuli-responsive behaviors. In this review, we summarized recent progress in the ultralong organic afterglow materials via host-guest strategy of small organic molecules, which can form various molecular interactions like hydrogen bonding interactions, π–π interactions and so on. The chemical structures of typical hosts and guests were summarized in Figs. 6 and 7. Besides, the photophysical properties were listed in Table 1 and the mechanisms of generated ultralong afterglow have been discussed in detail. Impressively, colorful ultralong afterglow materials, high-temperature ultralong afterglow materials, Ex-De ultralong afterglow materials, NIR ultralong afterglow materials and so on have been realized respectively. These materials have been successfully applied in advanced data encryption and anticounterfeiting, afterglow display and temperature sensing. This review not only highlights the recent developments of ultralong organic afterglow, but also provides a useful guiding principle on the design of excellent URTP, UTADF, and OLPL afterglow materials.

**Table 1:** Photophysical properties of ultralong organic afterglow materials

Host	Guest	Doping ratio	State	Afterglow wavelength [nm]	Afterglow lifetime [s]	Φ_Afterglow_	Refs.
BrPMe	Bd	1.2 mol%	amorphous	570, 620	0.11, 0.11^a^	-	^51^
DCB	NL	1 wt%	-	517	0.760	15.6%	^54^
TCB	NL	1 wt%	-	516	0.597	5.3%	^54^
DMB	NL	1 wt%	-	511	0.255	1%	^54^
TriCB	NL	1 wt%	-	508	0.203	1.5%	^54^
TPhE	Py	1 mol%	crystalline	~596^b^	0.4059	-	^55^
Cz	PyCz	0.5 wt%	crystalline	570, 770	0.373; 0.134	0.1%	^56^
Cz	BPCz	0.5 wt%	crystalline	695, 770	0.698; 0.672	2.2%	^56^
MCz	MPyCz	0.5 wt%	crystalline	695; 770	0.232; 0.214	4.9%	^56^
MCz	MBPCz	0.5 wt%	crystalline	695; 770	0.255; 0.217	3.3%	^56^
BA	CPBA	0.66 wt%	amorphous	430 (λ_ex_: 310); 536 (λ_ex_: 365)	1.97; 0.62	-	^57^
DTT	PCz	0.1 wt%	-	510	1.0771 ± 0.015	16.2%	^58^
*β*-estradiol	Ch1	3 wt%	amorphous	~475^b^	1.0	-	^59^
*β*-CD	*p*-Bph-BOH	33.3 mol%	aqueous	492	1.03	3.3%	^60^
(*S*)-H_8_-BINAP	(*S*)-BINAP	10 wt%	crystalline	~549^b^	0.44	3%	^61^
*β*-estradiol	DPAF	1 wt%	amorphous	~503^b^	1.19	6.2%	^61^
CB7	carboline 3	50 mol%	-	520	1.2	3%	^62^
CB7+*β*-CD	carboline 3	1:1:1	-	520	2.1	7%	^62^
DMB	PBA	0.5 mol%	crystalline	~520^b^	0.1207	5.1%	^63^
DMB	BBA	0.1 mol%	crystalline	~520^b^	0.1039	5.6%	^63^
DMB	BBA	0.5 mol%	crystalline	~520^b^	0.1258	6.3%	^63^
DMB	BBA	2 mol%	crystalline	~520^b^	0.1223	6.1%	^63^
DMB	DNapA	0.5 mol%	crystalline	~565^b^	0.1349	6.2%	^63^
DMB	DNPD	0.5 mol%	crystalline	~570^b^	0.1182	5.3%	^63^
TPA	TPA1	0.1 mol%	crystalline	525, 558	0.290	3.8%	^64^
TPP	TPA1	1 wt%	crystalline	496, 529, 561	0.104	25.5%	^64^
TPP	TPA2	1 wt%	crystalline	503, 513, 538	0.133	11.3%	^64^
SF	AD	0.1 wt%	crystalline	490	~0.380	1.19%	^65^
cyanuric acid	TMA	0.5 wt%	-	~406	1.13	9.3%	^66^
cyanuric acid	TMA, water	0.5 wt%, 20 wt%	-	~406	1.67	46.1%	^66^
BP	MAP1	-	-	572	0.2102	0.41%	^67^
DPOBP	NAPP	1 wt%	amorphous	~595^b^	0.106	-	^68^
DPOBP	PY	1 wt%	amorphous	~595^b^	0.202	-	^68^
DPOBP	RhB	1 wt%	amorphous	~600^b^	0.112	-	^68^
DPOBP-Br	NAPP	1 wt%	amorphous	~550^b^	0.156	-	^68^
DPOBP-Br	NAPM	1 wt%	amorphous	~550^b^	0.121	-	^68^
DPOBP-Br	PY	1 wt%	amorphous	~560^b^	0.166	-	^68^
TBBU	DTBU	0.1 mol%	crystalline	528, 566	0.340	5.9%	^69^
TBBU	DTBU	1 mol%	crystalline	528, 566	0.390	5.7%	^69^
TBBU	DTBU	10 mol%	crystalline	528, 566	0.422	4.8%	^69^
DTT	H1	0.1 mol%	-	511	1.0771	-	^70^
DTT	H2	0.1 mol%	-	534	0.5944	-	^70^
DTT	C	0.1 wt%	-	~570^b^	0.2690	4%	^71^
DTT	S	0.1 wt%	-	~570^b^	0.5615	18.7%	^71^
DTT	O	0.08 wt%	-	~570^b^	0.9817	27.6%	^71^
CA	TTCA	0.333 wt%	crystalline	490	2.86	1.76%	^72^
CA: MA = 3:1	TTCA	0.333 wt%	crystalline	490	3.21	7.31%	^72^
CA: MA = 1:1	TTCA	0.333 wt%	crystalline	489	3.16	5.60%	^72^
CA: MA = 1:3	TTCA	0.333 wt%	crystalline	488	2.92	4.68%	^72^
MA	TTCA	0.333 wt%	crystalline	497	0.43	0.65%	^72^
CA: BU = 3:1	TTCA	0.333 wt%	crystalline	489	3.30	3.69%	^72^
CA: BU = 1:1	TTCA	0.333 wt%	crystalline	489	3.36	5.17%	^72^
CA: BU = 1:3	TTCA	0.333 wt%	crystalline	488	3.19	3.95%	^72^
BU	TTCA	0.333 wt%	crystalline	485	2.88	2.55%	^72^
CA:UA = 3:1	TTCA	0.333 wt%	crystalline	489	3.32	3.47%	^72^
CA:UA = 1:1	TTCA	0.333 wt%	crystalline	489	3.16	3.34%	^72^
CA:UA = 1:3	TTCA	0.333 wt%	crystalline	490	2.80	2.12%	^72^
UA	TTCA	0.333 wt%	crystalline	489	0.24	0.08%	^72^
CA	2-A	1 wt%	crystalline	430, 460	0.800	76.42%	^73^
CA	4-A	1 wt%	crystalline	500	0.690	39.56%	^73^
CA	5-A	1 wt%	crystalline	480	0.790	23.01%	^73^
CA, MA	1-NA	16.7 wt%	aqueous	548	0.2196	-	^74^
CA, MA	2-NA	16.7 wt%	aqueous	527	0.4931	-	^74^
CA, MA	BDA	-	aqueous	526	0.3303	-	^74^
CA, MA	TPA	-	aqueous	~440^b^	0.4415	-	^74^
CA, MA	1-OH-2-NA	-	aqueous	~526^b^	0.2398	-	^74^
CA, MA	1-OH-4-SO_2_NH_2_-2-NA	-	aqueous	~515^b^	0.3709	-	^74^
F-Ph-L	F-Na-L	0.1 wt%	-	526	0.637	-	^75^
F-Ph-D	F-Na-L	0.1 wt%	-	526	0.322	-	^75^
F-Ph-D	F-Na-D	0.1 wt%	-	526	0.649	-	^75^
F-Ph-L	F-Na-D	0.1 wt%	-	526	0.454	-	^75^
F-Ph-L	F-Na-DL	0.1 wt%	-	526	0.986	-	^75^
F-Ph-D	F-Na-DL	0.1 wt%	-	526	0.968	-	^75^
BP	DBAC-BA	0.2 mol%	crystalline	570	0.261	5.10%	^76^
BP	DBAC-Br	0.2 mol%	crystalline	571	0.199	12.8%	^76^
BP	DBAC	0.2 mol%	crystalline	560	0.330	8.74%	^76^
TPP	DBAC-BA	0.2 mol%	crystalline	550	0.322	13.3%	^76^
TPP	DBAC-Br	0.2 mol%	crystalline	556	0.236	9.6%	^76^
TPP	DBAC	0.2 mol%	crystalline	550	0.375	7.3%	^76^
TPA	DBAC-BA	0.2 mol%	crystalline	530	0.151	7.03%	^76^
TPA	DBAC-Br	0.2 mol%	crystalline	530	0.111	9.2%	^76^
PPy	PF-Np	0.1 mol%	crystalline	563, 610	0.249	5.9%	^77^
PPy	PF-Pe	0.1 mol%	crystalline	562	0.565	19.4%	^77^
PPy	PF-Py	0.1 mol%	crystalline	615, 669	0.217	8.4%	^77^
PPy	PF-Pe, EB	0.1 mol%,0.2 mol%	-	620	0.252	-	^77^
PPy	PF-Pe, EY	0.1 mol%,0.1 mol%	-	605	0.286	-	^77^
Host-CS	G-CS	10 wt%	crystalline	650	0.492	-	^78^
CPAN	OTC	0.05 wt%	crystalline	536	0.4835	16.40%^c^	^79^
DPAN	OTC	0.05 wt%	crystalline	545	0.2865	1.02%^c^	^79^
BA	OHA	0.033 wt%	amorphous	400	0.73	-	^80^
BA	MHA	0.033 wt%	amorphous	437	1.85	-	^80^
BA	PHA	0.033 wt%	amorphous	420	2.01	-	^80^
BA	FL	0.05 wt%	amorphous	488, 550	~0.5^b^	~25%^b^	^81^
BA	PAC1	0.1 wt%	crystalloid	406, 472, 500, 528	1.1229; 1.7145; 1.6190;1.4059	8.47%	^82^
BA	PAC2	0.1 wt%	crystalloid	365, 460, 492, 544	1.8118; 1.9896; 1.9480;1.9192	10.93%	^82^
BA	PAC3	0.1 wt%	crystalloid	406; 478; 512; 544	0.9810; 0.9906; 0.9556; 0.9381	9.95%	^82^
BA	PAC4	0.1 wt%	crystalloid	416, 484, 520, 556	1.8512; 2.1701; 2.2146; 2.1077	15.87%	^82^
BA	PAC5	0.1 wt%	crystalloid	455, 528, 560	0.7917; 1.1248; 1.1268	10.39%	^82^
BA	PAC6	0.1 wt%	crystalloid	464, 536, 570, 612	0.5608; 0.9166; 0.8984;0.8593	8.35%	^82^
BA	PAC7	0.1 wt%	crystalloid	514, 546, 586	2.1239; 2.1697; 2.3185	7.24%	^82^
BA	PAC8	0.1 wt%	crystalloid	486; 554; 590	0.4080; 0.5504; 0.5697	7.03%	^82^
BA	PAC9	0.1 wt%	crystalloid	528, 648, 708	0.5542; 0.1706; 0.1679	5.01%	^82^
BA	PAC10	0.1 wt%	crystalloid	560, 648, 710	0.3185; 0.1898; 0.1841	3.91%	^82^
BA	PAC11	0.1 wt%	crystalloid	466, 534,568	0.5656; 1.0042; 0.9597	14.21%	^82^
BA	PAC12	0.1 wt%	crystalloid	476; 540; 576	0.6731; 1.0260; 1.0480	11.97%	^82^
Cz	Bd	0.05 wt %	crystalline	393, 565	0.038, 0.355	0.92%	^83^
Cz	BCz	1.0 wt %	crystalline	426; 547	0.058; 1.036	2.03%	^83^
Cz	DBCz	0.5 wt %	crystalline	458; 550	0.632; 0.748	1.66%	^83^
CBP-2CN	CBP-Bd2CN	1 mol%	-	430; 560	0.158, 0.369	-	^84^
CBP-CN	CBP-Bd2CN	1 mol%	-	450; 560	0.122, 0.136	-	^84^
CBP	CBP-Bd2CN	1 mol%	-	452; 560	0.064, 0.110	-	^84^
TPA	TPA-DPPQ	0.1 mol%	-	570	0.1667	-	^85^
TPA	TPA-PTPQ	0.1 mol%	-	516; 605	0.0076 (7.2%),0.1079 (92.8%);0.1475	-	^85^
TCN-H	BTDA-H	1.0 wt %	crystalline	410; 490	1.056, 1.027	2.6%; 10.7%	^86^
TCN-F	BTDA-F	1.0 wt %	crystalline	410; 495	0.986, 0.923	0.27%,10.03%	^86^
TCN-Cl	BTDA-Cl	1.0 wt %	crystalline	410; 500	0.531, 0.442	0.14%, 14.86%	^86^
TCN-Br	BTDA-Br	0.01 wt %	crystalline	510	0.305	24.1%	^86^
POOH	NDOH	0.1 mol%	crystalline	610	0.1271	1.6%	^87^
PBOH	NDOH	0.1 mol%	crystalline	614	0.1590	3.9%	^87^
PMOH	NDOH	0.1 mol%	crystalline	580	0.1676	3.8%	^87^
PHOH	NDOH	0.1 mol%	crystalline	589	0.1650	2.1%	^87^
PBOH	NDH	0.1 mol%	crystalline	620	0.1383	2.1%	^87^
PBOH	NDBr	0.1 mol%	crystalline	612	0.1050	4.9%	^87^
PBH	NDH	0.1 mol%	crystalline	634	0.1425	2.6%	^87^
PBH	NDOH	0.1 mol%	crystalline	630	0.1299	1.9%	^87^
PBH	NDBr	0.1 mol%	crystalline	585	0.1136	1.1%	^87^
PBBr	NDOH	0.1 mol%	crystalline	590	0.1021	2.0%	^87^
PBBr	NDBr	0.1 mol%	crystalline	590	0.1058	5.1%	^87^

^a^Measured in vacuum

^b^Approximate numbers determined from reference

^c^TADF quantum yields

Future challenges and areas of focus for ultralong organic afterglow materials can be divided into the following aspects. Firstly, the precise manipulation of luminescent properties of URTP materials with high efficiency is demanded for real-life applications, which will broaden the application of these URTP materials in ultrafine optical data storage and information encryption, bioimaging with drug transportation, chiral light-emitting transistor, and so on. It is expected to achieve ultra-small size and large capacity with more complex and higher-level of time-solved optical information storage. Moreover, achieving NIR emission with an ultralong lifetime is still challenging for practical applications. Secondly, traditional organic compounds have been rarely reported for the design of pure UTADF with high efficiency, which could be a hot spot of research to be further enriched for their potential applications in high-temperature fine imaging, etc. Third, strategies for systematic design of OLPL materials need more research and advanced techniques need to be more explored to directly prove the luminescence mechanism for future research. Finally, efficiency limitations arising from competitive non-radiative decay pathways and environmental/thermal stability challenges in most amorphous organic systems are always major obstacles to practical applications. As a result, future research should prioritize the development of organic ultralong afterglow materials with high efficiency, robust thermal stability, and excellent environmental compatibility. It is expected that the review will provide valuable insights and strategies for the development of high-performance ultralong organic afterglow materials.
